# Dams Determine the Composition and Activity of Microbial Communities in Semiclosed Marine Basins of the White and Barents Seas, Russia

**DOI:** 10.3390/microorganisms13092143

**Published:** 2025-09-12

**Authors:** Alexander S. Savvichev, Nikolay A. Demidenko, Vitaly V. Kadnikov, Alexey V. Beletsky, Valeria V. Belenkova, Igor I. Rusanov, Pavel A. Sigalevich, Daria A. Ivanova

**Affiliations:** 1 Winogradsky Institute of Microbiology, Research Center of Biotechnology, Russian Academy of Sciences, Moscow 117312, Russia; 2 Shirshov Institute of Oceanology, Russian Academy of Sciences, Moscow 117997, Russia; 3 K.G. Skryabin Institute of Bioengineering, Research Center of Biotechnology, Russian Academy of Sciences, Moscow 117312, Russia; vkadnikov@bk.ru (V.V.K.);

**Keywords:** microbial communities, microbial processes, tidal power stations, meromictic basins, sulfide poisoning

## Abstract

Microbiological and biogeochemical investigation of the bottom sediments of semiclosed basins was carried out at the Kislaya Guba tidal power station (Barents Sea) and in Kanda Bay (White Sea). Suppressed tidal water mixing is known to affect the hydrological regime of isolated basins, resulting in the development of oxygen-free sediments. The upper sediments of the studied bays were shown to contain higher concentrations of sulfide and methane, with increased rates of sulfate reduction, methanogenesis, and methane oxidation. The relative abundance of truly marine microorganisms decreased, while microorganisms common in anoxic sediments of meromictic basins developed. The indicator microorganisms with increased relative abundance were archaea of the genera *Methanoregula* and *Methanosaeta*. Bacteria of the class Chlorobia, Chloroflexi of the family *Anaerolineaceae*, and *Rhodoferax*-related bacteria were indicators of the stagnant seawater. Members of the genus *Woeseia* were counter-indicators, occurring only in marine water. In our opinion, under reasonably regulated water exchange via the dams, the ecosystems of the Kanda and Kislaya Guba bays may retain the characteristics of marine bays. Otherwise, the studied bays may become stratified basins with anoxic near-bottom water, harboring microbial communities similar to those inhabiting meromictic basins.

## 1. Introduction

The coasts of the Barents and White seas are characterized by highly irregular coastlines, with numerous elongated bays and fjords [1]. They are also known for active tidal processes, with diurnal fluctuations of 2 m or more. The tidal currents are responsible for the constant water exchange in bays and fjords, preventing the development of stagnant, anoxic waters.

Semiclosed marine basins have high biological productivity and harbor considerable amounts of economically important plants and animals. Seacoast development, such as the construction of embankments, dams, weirs, and tidal power stations, inevitably leads to the closure of bays. Shutting off the estuaries could lead to a decline in migratory fish populations, potentially causing their disappearance, and it may restrict their range. In industrial areas, partial isolation of a sea basin results in elevated pollutant levels in the water [2]. In the case of critically decreased water exchange in regulated basins, irreversible damage to the ecosystems is possible, with complete loss of the biological resources (macrophytes, mollusks, fish, etc.). Due to rapid changes in artificial sea basins, they can serve as models for reconstructing different stages of changes in coastal ecosystems when natural isolation of semiclosed basins from the sea increases [3].

The composition of prokaryotic communities of the water column and bottom sediments of marine bays and fjords depends on the hydrological regime of these basins. Regular mixing of the water column due to wind, tides, and seasonal processes results in the flow of oxygen-containing water and, therefore, in the formation of an oxygen-dependent microbial community. On the contrary, decreased water exchange results in oxygen depletion and the formation of anoxic zones with a noticeably different microbial community composition. Microbial communities in anoxic water differ significantly from those in oxygen-containing water columns [4,5,6,7]. When light penetrates to the upper boundary of the anoxic zone, the microbial community is predominated by anoxygenic phototrophic bacteria (APB). In the absence of light, microbial chemosynthesis is activated [8,9,10,11,12,13].

The rates of microbial processes and the structure of communities responsible for them were investigated at two artificially separated bays of the Barents and White seas. The Kislaya Guba Bay is separated from the open Barents Sea by the dam of a tidal power station (TPS). The environmental impacts associated with the construction of TPS Kislaya Guba were studied and presented in a monograph [14]. However, no microbiological studies were carried out at this early stage. The Kanda Bay is separated from the White Sea by a railway dam. We have previously conducted studies on the activity of microbial processes in the waters of the Kanda Bay [15]. Our goal was to obtain data on the hydrochemical parameters, microbial community composition, and the rates of the processes of the carbon and sulfur cycles in the upper bottom sediments of the Kanda and Kislaya Guba bays.

The significance of studying microbial communities and processes in basins separated from the main sea basin is related to predicting the negative effects of sulfide contamination in such artificial reservoirs and the development of a near-bottom anoxic zone; the latter is a global trend occurring in all oceans [15,16,17].

## 2. Materials and Methods

### 2.1. Characteristics of the Investigation Objects

**Figure 1:**
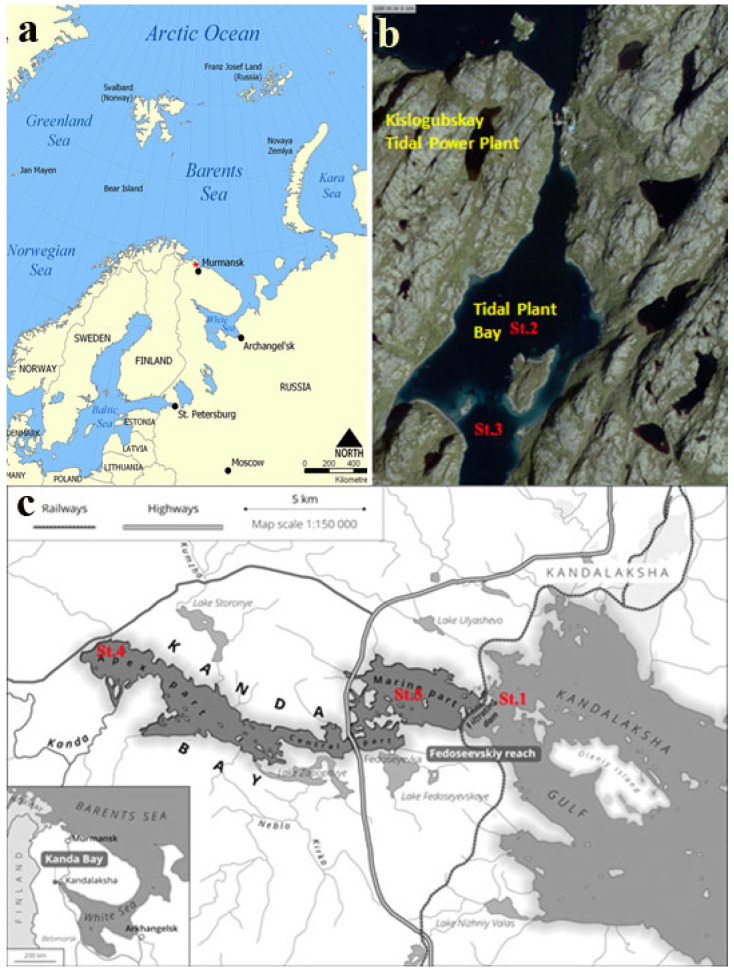
Map of the Barents Sea coast. The red marker indicates the location of the tidal power plant (**a**); Kislaya Guba map (satellite https://nakarte.me (accessed on 3 July 2025), Yandex Satellites) (**b**), and schematic map of the Kanda Guba Bay (**c**). The station numbers (Sts. 1, 2, 3, 4, and 5) are marked in red.

The basin of the Kislaya Guba tidal power station (TPS) is a sea bay (Kislaya Guba) with a narrow entrance and an underwater threshold (Figure 1). Two depressions (36 and 60 m) are located in the central and apex parts of the bay. Before TPS construction (in 1965–1968), there was free water exchange with the sea. The first and only TPS in Russia commenced operation in 1968. The tidal flow regime in the area was heavily impacted during TPS construction and operation, leading to the development of a stagnant, oxygen-depleted near-bottom water layer and sulfide poisoning. The Kislaya Guba TPS ceased operations in the 1990s; subsequently, the flow regime was partially recovered. Currently, there is limited water exchange between the Kislaya Guba Bay and the open sea waters.

The Kanda Bay in the western part of the Kandalaksha Gulf (White Sea) has a complex, sinuous shape. This is an artificial basin, with a complex shoreline, which was separated from the Kandalaksha Gulf by a railway dam since 1916. The Kanda River and numerous springs flow into the bay. The railway crossing the Kanda Bay was under construction from September 1915 to November 1916. The filtration dam was constructed at the shallow part of the sea. Large boulders were positioned at the base of the construction, while smaller boulders and rocks were used for the upper part. As a result, tidal water could penetrate the dam. A single-span bridge was constructed to provide passage for fish and small vessels.

### 2.2. Main Idea and Scheme of Research

Samples were collected at six stations in the bays of the White and Barents seas, including two reference (control) ones. One of the latter (St. 1) was located in the marine part of the Kandalaksha Gulf, White Sea. According to our hypothesis, the microbial community at the upper sediments of this station reflected the initial stage, prior to the bay isolation. St.6 was at the meromictic Lake Trekhtzvetnoe (Kandalaksha Galf, White Sea), which became separated from the sea bay in the early 20th century due to natural processes of land raising [18,19]. We hypothesized that the sediment community at this station to represent the climax state of a microbial community existing at highly reducing conditions. Four experimental stations (Sts. 2, 3, 4, and 5) were located in the isolated parts of the Kanda and Kislaya Guba bays. These isolated basins were to various extents affected by seawater, which replenishes and mixes the water column during the tides (Figure 1).

### 2.3. Sampling and Sample Processing

The samples of water and bottom sediments were collected in winter–spring 2023–2024 through the ice holes. Temperature and dissolved oxygen concentration were determined with an HI8314F portable electric thermometer/oxymeter (Hanna Instruments, Vöhringen, Germany). Salinity was measured with an HI8733 conductometer (Hanna Instruments, Vöhringen, Germany). Water was sampled with a 1 L limnological bathometer attached to a calibrated cable. Sediment samples were collected with a limnological stratometer with a glass tube 60 mm in diameter. The samples were stored and transported at 4 °C. For sulfide determination, the water samples were immediately fixed with zinc acetate and then analyzed using N,N-dimethyl-*p*-phenylenediamine.

To determine the total microbial abundance (MA), the samples of near-bottom water were fixed with glutaraldehyde at a final concentration of 2%, filtered through black polycarbonate membranes (Millipore), and stained with Acridine orange. The filters were examined under an epifluorescence microscope (Axio Imager D1, Carl Zeiss, Oberkochen, Germany) equipped with a visualization system (Image Scope Color, Leica Biosystems, Nussloch, Germany) at a magnification of ×1000. To calculate the biomass based on the measured volume of microbial cells, a cell density of 1.0 mg mm^−3^ was assumed for wet biomass, and specific biomass (B) was presented in micrograms per liter (µg L^−1^). Single cells and cell aggregates in stained preparations were enumerated separately. Cell groups with a common outline, in which individual cells were difficult or impossible to identify and count, were considered aggregates.

Methane content was determined using the head-space sampling method. Methane concentration was measured on a Kristall-2000-M gas chromatograph (Meta-Chrom, Yoshkar-Ola, Russia) with a flame ionization detector, with the measurement error not exceeding ±5%. Pore water was obtained from the sediments using centrifugation at 5000× *g* on a TsUM-1 centrifuge (Moscow, USSR). Alkalinity was determined with the relevant reagent kit (Merck Group, Darmstadt, Germany). Sulfate and chloride concentrations in pore water were determined on a Staier ion chromatograph (Akvilon, Podolsk, Russia).

### 2.4. Rates of Microbial Processes

The rates of dark CO_2_ assimilation (DCA), hydrogenotrophic methanogenesis (MGh), methane oxidation (MO), and sulfate reduction (SR) were determined by radiotracer analysis with ^14^C- and ^35^S-labeled substrates. For this purpose, bottom sediment samples (2.5 cm^3^) were collected into cut-off 5 mL plastic syringes and sealed with butyl rubber stoppers. The labeled substrates (0.2 mL) were injected through the stopper with a tuberculin syringe and distributed uniformly along the syringe length. The rate of methane oxidation was determined using ^14^C-labeled methane dissolved in sterile distilled water (1 μCi per sample). The rate of sulfate reduction was quantified using ^35^S-labeled sulfate at a dose of 2.5 μCi per sample. The rates of methanogenesis and microbial CO_2_ assimilation were determined using ^14^C-labeled bicarbonate (4.0 μCi per sample). After 24 h incubation at a temperature close to in situ values, the samples were fixed with 1.0 mL of 1 M KOH. The samples fixed in this manner prior to the addition of the labeled substrates were used as the controls. Subsequent sample processing and calculation of the rates of microbial processes were carried out as described previously [20,21]. Radioactivity (^14^C and ^35^S) of the products of the studied processes was measured on a PackardTRI-CarbTR 2400 liquid scintillation counter (PerkinElmer, Shelton, USA). The rates of microbial processes (DCA, MGh, MO, and SR) were calculated using the averages for two replicate measurements per sample.

### 2.5. Isolation of Metagenomic DNA, PCR Amplification, and High-Throughput Sequencing of the 16S rRNA Gene Fragments

Sediment samples in sterile 2 mL tubes were transported to the laboratory at a temperature of 2–4 °C. The samples were homogenized by grinding with liquid nitrogen, and the metagenomic DNA preparation was prepared using the Power Soil DNA Isolation Kit (MO BIO Laboratories, Carlsbad, CA, USA). The composition of the prokaryotic community was determined by analyzing the sequences of the V3–V4 variable region of the 16S rRNA gene, amplified by PCR using the primers PRK341F (5′-CCTACGGGRBGCASCAG-3′) and PRK806R (5′-GGACTACYVGGGTATCTAAT-3′) [22]. The obtained PCR fragments were used to construct the library using the Nextera XT DNA Library Prep Kit (Illumina, San Diego, CA, USA) according to the manufacturer’s protocols. The libraries were sequenced on MiSeq (Illumina, San Diego, CA, USA) using MiSeq Reagent Kit V3 (in paired reads format, 2 × 300 nt). The obtained reads were combined using the FLASH v 1.2.11 software package [23]. A total of 48,153 sequences of the 16S rRNA gene fragments were obtained per sample. Each set of sequences was clustered into operational taxonomic units (OTUs) at 97% identity; the chimeras were removed with Usearch [24]. Alpha diversity indices were calculated based on an OTU frequency table using Usearch.

A 16S phylogenetic tree was inferred using FastTree v2.1.11 from the alignment constructed by MAFFT v7.055b. Default parameters were used for both programs, and local support values were computed with the Shimodaira–Hasegawa test. Taxonomic identification of the OTUs was performed using SINA with the SILVA database and default parameters [25]. All obtained sequences were deposited in the NCBI Sequence Read Archive (SRA) under project PRJNA986318.

## 3. Results

### 3.1. Physicochemical Characterization of the Samples

Physicochemical and biogeochemical characteristics of the reference and experimental samples are listed in Table 1.

**Figure 2:**
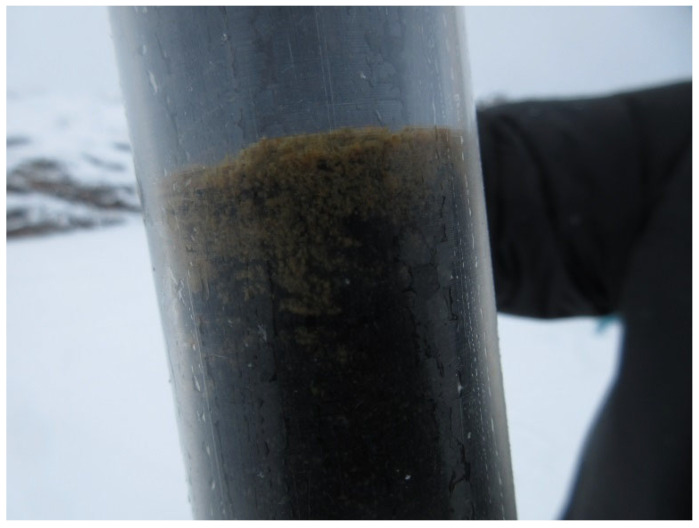
Bottom sediment core from the Kislaya Guba TPS bay. The upper (0–2 cm) and lower (2–12 cm) layers are clearly visible.

The color and consistency of the sediments were diverse. Sample 1 (“marine”) consisted of aleurite and fine sand. Sample 2, carefully collected from the top of the sediment sample (0–2 cm), consisted of loose brown flakes; black pelite–aleurite sediment was located below, at 2–7 cm (sample 3). The boundary between these layers was rather well discernible (Figure 2).

Sample 4 consisted of a semiliquid, black, flaky sediment with inclusions of plant material. Sample 5 consisted of black, slightly compacted pelite. These two samples had a distinct odor of hydrogen sulfide. At St. 6 (“anoxic”), the sediment was black, semiliquid pelite with a strong smell of sulfide.

Due to the sampling time (winter) and procedure (through ice holes), the surface water was cold (close to 0 °C). The sediments and near-bottom water were warmer, from 1.0 to 4.8 °C for the Kandalaksha Gulf open part and the Kanda Bay Fedoseevskyi reach, respectively. Water salinity varied as well, from 13.5‰ in the Kanda Bay apex part to 33.5‰ in the Kislaya Guba TPS bay. The redox potential of samples 1 and 2 was pronouncedly positive. The Eh value of sediment 3 was unstable and close to zero, indicating the boundary redox zone. The sediments of samples 4–6 were pronouncedly reduced and contained sulfide. The concentration of dissolved methane in sample 1 was very low (0.64 µmol). In the Kislaya Guba sediments, methane concentration increased to 12–28 µmol, while in sulfide-containing Kanda Bay sediments, it varied from 45 to 158 µmol, reaching 2220 µmol in sediment 6. Thus, methane concentration in the studied sediments varied 3500-fold.

### 3.2. Rates of Microbial Processes in the Upper Sediment Layers

During sampling, photosynthetic activity (up to 1.5 µmol C L^−1^ day^−1^) was detected only in the narrow water layer immediately below the ice. In lower horizons, photosynthetic activity was not reliably detected. Dark CO_2_ assimilation (DCA) is an integral parameter, combining the values for both heterotrophic carboxylation and autotrophic CO_2_ consumption via chemosynthesis. Low DCA rates were observed in the sediments of the “marine” St. 1 (90–110 nmol dm^−3^ day^−1^). In the Kislaya Guba sediments (St. 2 and St. 3), the DCA was higher (0.2–1.0 µmol dm^−3^ day^−1^). Still higher DCA rates were revealed in the Kanda Bay reduced sediments of St. 4 and St. 5 (3–7 µmol dm^−3^ day^−1^). The highest rates of microbial processes were shown for sulfide-containing sediments of St. 6 (28–48 µmol dm^−3^ day^−1^). In marine sediments at St. 1, methanogenesis rates (MGh) were below the reliable detection limit of 2 nmol L^−1^ day^−1^; however, since only the rate of hydrogenotrophic methanogenesis H_2_/CO_2_ as the substrate was actually measured, these values may be underestimates. The MGh rate was considerably higher in the Kislaya Guba sediments (3–10 nmol dm^−3^ day^−1^). Still higher MGh rates were found in the sediments of the Kanda Bay (8–40 nmol dm^−3^ day^−1^) and of the meromictic lake (90–130 nmol dm^−3^ day^−1^). The rates of methane oxidation (MO) increased from 50–90 nmol dm^−3^ day^−1^ at St. 1 to 1.8 µmol dm^−3^ day^−1^ at St. 6. The sulfate reduction (SR) rate was low in the sediment of St. 1 (10–30 nmol S dm^−3^ day^−1^), higher at Sts. 2 and 3 (0.5–1.2 µmol S dm^−3^ day^−1^), and high (8–20 µmol S dm^−3^ day^−1^) in the sediments of St. 4 and St. 5. As was expected, the highest SR rate was revealed in the sulfidic sediment of St. 6 (up to 68 µmol S dm^−3^ day^−1^).

### 3.3. Total Microbial Abundance

Microscopy of the water samples revealed that microbial abundance (MA) in near-bottom water of St. 1 was 200 ± 50 ×·10^3^ cells mL^−1^. MA in the near-bottom water of the Kislaya Guba and Kanda bays was considerably higher (on average, 480 ×·10^3^ cells mL^−1^). In the near-bottom water of St. 6, MA was still higher (up to 12 ×·10^6^ cells mL^−1^). Microbial biomass varied from 0.2 mg L^−1^ at the marine St. 1 to 4800 mg L^−1^ in Lake Trekhtzvetnoe (St. 6).

### 3.4. Results of High-Throughput Sequencing

The taxonomic composition of microbial communities in the upper sediments of the Kanda and Kislaya Guba bays, as well as at two reference stations, was determined using high-throughput sequencing of the 16S rRNA gene fragments (Table 2).

To achieve this goal, a total of 161,472 16S rRNA gene fragments were identified. These sequences were clustered, yielding 3554 bacterial and 1692 archaeal OTUs at 97% identity. Archaea constituted 13.8 to 39.8% of all 16S rRNA gene sequences (Table 2). Analysis of the studied samples revealed bacteria and archaea belonging to 69 phyla. For 24 phyla, the OTU number exceeded 1% in at least one of the samples. They were identified using SILVA according to the GTDB database: *Nanoarchaeota*, *Proteobacteria*, *Chloroflexi*, *Bacteroidota*, *Verrucomicrobiota*, *Planctomycetota*, *Desulfobacterota*, *Actinobacteriota*, *Acidobacteriota*, *Firmicutes*, *Patescibacteria*, *Latescibacterota*, *Thermoplasmatota*, *Spirochaetota*, *Myxococcota*, *NB1-j*, *Cyanobacteria*, *Zixibacteria*, *Crenarchaeota*, *Halobacterota*, *Campylobacterota*, *Caldatribacteriota*, *Acetothermia*, and *Thermotogota*.

According to the Chao1 index, the lowest species richness (789.5) was found in the marine oligotrophic community of St. 1. In the sulfidic sediment of St. 6, the species richness was also low (873.5). These low values resulted from oligotrophic conditions of the water column in the first case and the limiting effect of high sulfide concentrations in the second case. In the Kanda Bay (St. 4 and St. 5), the species richness was higher (1471–1568). The highest species richness was revealed in the subsurface sediment at the tidal power station bay (1606; St. 3).

The Shannon *H’* (log with base *e*) values were rather uniform, with the minimum (4.63) for the marine station (St. 1) and the maximum (5.98) for the sediments of the tidal power station bay (St. 3).

### 3.5. Taxonomic Composition of the Sediment Microbial Communities at the Phylum Level

**Figure 3:**
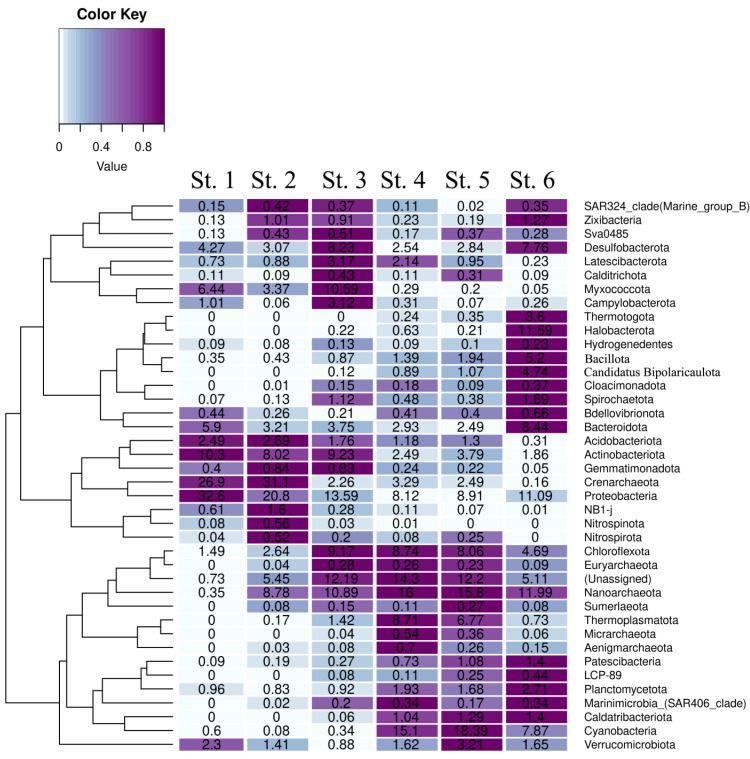
Taxonomic composition (at the phylum level) of microbial communities of the upper sediment from stations at the Barents and White seas.

The 24 phyla listed above were subdivided into three groups. The first one comprised the phyla most numerous in the sediments of the marine St. 1, while they were absent or much less abundant in the sulfidic sediment of St. 6. These were mostly *Crenarchaeota archaea* and bacterial phyla *Pseudomonadota*, *Actinobacteriota*, *Myxococcota*, and *Acidobacteriota*. The second group comprised the phyla exhibiting the opposite tendency. The OTUs of members of these phyla were most abundant in the sulfidic sediment of St. 6 and were absent or constituted less than 1% in the sediments of the marine station St. 1 (Figure 3). These were archaea *Nanoarchaeota* and *Halobacterota*, as well as bacteria *Bacillota*, *Candidatus Bipolaricaulota* (heterotypic synonym Acetothermia), *Thermotogota*, *Planctomycetota*, *Spirochaetota*, *Caldatribacteriota*, and *Patescibacteria*.

In the sediment communities from the Kanda and Kislaya Guba bays, the phyla belonging to these two groups were generally less abundant than at the reference St. 1 and St. 6 (Figure 3). The third group comprised the phyla most represented in the sediments of the Kanda and Kislaya Guba bays, with their OTU amounts at these stations exceeding those at Sts. 1 and 6. These were *Cyanobacteriota* (*Cyanobacterota*), *Desulfobacterota*, *Chloroflexota*, *Verrucomicrobiota*, *Thermoplasmatota*, *Campylobacterota*, and *Latescibacterota*. Members of these phyla were the indicators of an intermediate state of the sediments, from oxidized oligotrophic to highly reduced sulfidic ones.

### 3.6. Taxonomic Composition of the Sediment Microbial Communities at the Family Level

Family-level analysis of the composition of microbial communities made it possible to reveal the microbial taxa, indicating the changes associated with the isolation of the marine basin previously connected to the ocean.

**Figure 4:**
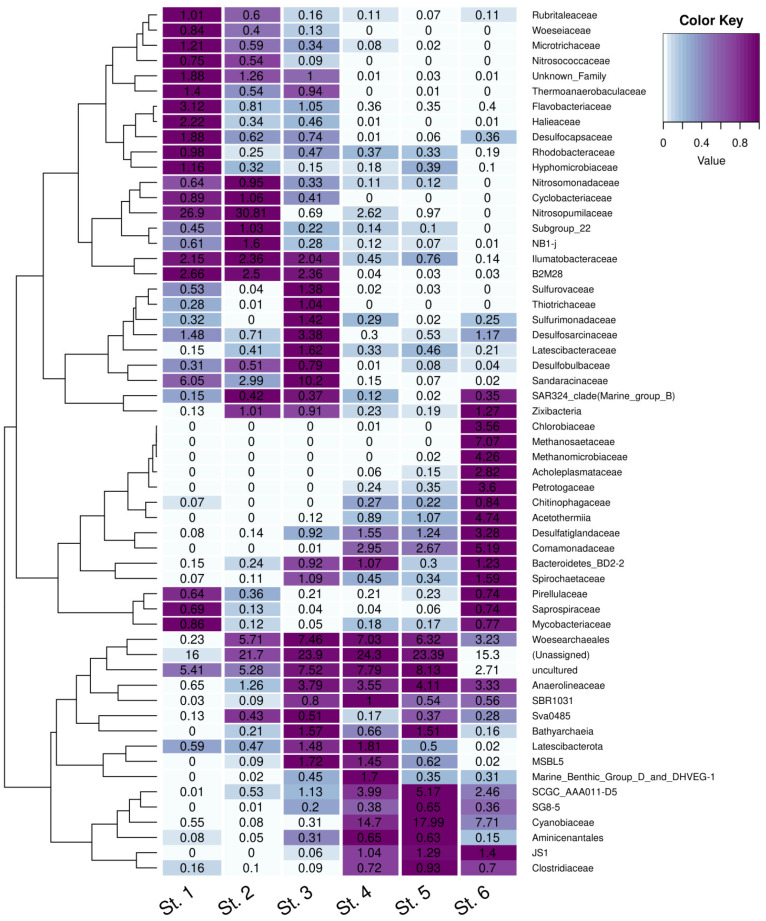
Taxonomic composition (at the family level) of microbial communities of the upper sediment from stations at the Barents and White seas.

The first group comprised the families most represented in the sediment of the marine St. 1 and absent or much less abundant in the sulfidic sediment of St. 6 (Figure 4).

In the archaeal community of the oxidized sediment at marine St. 1, uncultured members of the family *Nitrosopumilaceae*, phylum *Crenarchaeota*, were predominant (26.9% from all prokaryotes). Archaea of the family *Nitrosopumilaceae* are obligate aerobes able to oxidize ammonium, occurring in seawater at very low concentrations. They are hypothesized to be capable of autotrophic growth. Members of this family are found in both the photic zone of the sea and in the upper horizons of marine sediments worldwide [26,27,28]. Characteristically, in the sediment of St. 2, members of the family *Nitrosopumilaceae* constituted 30.8%, indicating positive changes due to the restoration of the flow regime in the TPS basin. The relative abundance of *Nitrosopumilaceae* in the sediments of other stations was very low; none were detected in the sulfidic sediment of St. 6.

Among bacteria, the organisms found in marine sediments were uncultured *Sandaracinaceae* (phylum *Myxococcota*) (St. 1, 6.05%; St. 3, 10.2%), as well as heterotrophic aerobic bacteria decomposing plant residues [29].

Uncultured *gammaproteobacterium* B2M28 (phylum *Pseudomonadota*) was revealed in the St. 1 sediment (2.66%). In other samples, they were present in lower numbers. Members of B2M28 have been found in the coastal sediments of various seas [30].

In the sediments of Sts. 1, 2, and 3, uncultured members of the family *Woeseiaceae* (phylum *Pseudomonadota*) were found. This is a widespread group of *Gammaproteobacteria*, which was originally named JTB255-Marine Benthic Group. These are typical inhabitants of deep-water marine sediments. A comparative genomic analysis indicated that members of this clade are likely to utilize proteinaceous material from cell walls and other organic residues present in marine sediments [31,32]. The only member of this family obtained in pure culture is probably *Woeseia oceani* from the Yellow Sea tidal bay [31].

Members of the family *Halieaceae* (clade OM60/NOR5, phylum *Pseudomonadota*) were revealed at Sts. 1, 2, and 3. Members of the phylum *Pseudomonadota* have been isolated from the coastal areas of various seas. Their massive growth usually follows phytoplankton blooms. The cultured *Halieaceae* possess the genes for proteorhodopsin [33].

Members of the family *Hyphomicrobiaceae* (phylum *Pseudomonadota*) also fell into the first group. Many *Hyphomicrobiaceae* species are oligocarbophilic, meaning that they exist only at low concentrations of carbon sources and are incapable of growth on rich media. Most of them are obligate chemoheterotrophs. Members of this family can be found in soils, freshwater lakes and springs, and marine environments [34].

Members of the family *Nitrosomonadaceae* (phylum *Pseudomonadota*) were found in the sediments of all stations, except for the sulfidic St. 6. All cultured *Nitrosomonadaceae* are lithoautotrophic ammonium oxidizers. Ammonium oxidizers control nitrification, oxidizing ammonium to nitrite, which is subsequently oxidized to nitrate by specialized bacteria. They, therefore, play a crucial role in regulating the nitrogen cycle in freshwater and marine basins [35].

Group II comprised the families predominant in the community of sulfidic sediment at St. 6, while they are absent or occur at minor amounts in the community of marine St. 1.

These were, first of all, archaea of the families *Methanomicrobiaceae* and *Methanosaetaceae* of the genera *Methanoregula* and *Methanosaeta* (phylum *Halobacterota*). The best-known species is *Methanoregula boonei*, originally isolated from a freshwater bog. Its metabolism is based on hydrogenotrophic methanogenesis [36]. Archaea of the genus *Methanosaeta* are halotolerant organisms widespread in marine environments; they use acetate for methanogenesis. The acetoclastic methanogen *Methanosaeta pelagica* has been isolated from the tidal zone of the Gulf of Tokyo [37]. No methanogenic archaea were found in the sediments of stations 1–4. At St. 6, methanogens were clearly dominant (4.26 and 7.07% for *Methanomicrobiaceae* and *Methanosaetaceae*, respectively; Figure 4).

In sulfidic sediments of St. 6, the predominant bacteria belonged to the genus *Rhodoferax*, family *Comamonadacea,* phylum *Pseudomonadota* (5.19%). These are nonsulfur purple bacteria capable of anoxygenic photosynthesis, although they are capable of growth in the dark. Apart from St. 6, *Rhodoferax* was found at the Kanda Bay stations 4 and 5 but not in the sediments of Sts. 1–3.

Bacteria of the family *Acetothermia* (*Candidatus Bipolaricaulota*; heterotypic synonym *Acetothermia*) also belonged to group II. They were found in the sediments of Sts. 4, 5, and 6 (0.89, 1.07, and 4.74%, respectively; Figure 4) and were absent from the sediments of Sts. 1 and 2. Members of the family *Acetothermia* are ubiquitous and have been found in various anaerobic habitats. Their physiology and ecological role are not well understood. Genome annotation and metabolic reconstruction indicate anaerobic chemo-heterotrophic metabolism, i.e., these bacteria grow by the fermentation of peptides, amino acids, and sugars, producing acetate, formate, and hydrogen [38].

Members of the family *Petrotogaceae* (*Thermotogota*) were revealed in the sediments of stations 4, 5, and 6 (3.69%) but not in the communities of stations 1 and 2. Mesophilic *Petrotoga* (unlike thermophilic *Thermotoga*) thrives by fermentation of various compounds [39]. Its trophic and ecological role in anoxic marine sediments remains unclear.

The presence of green sulfur bacteria of the family *Chlorobiaceae* (phylum *Bacteroidota*) is a reliable indicator of anoxygenic photosynthesis, with sulfide consumption occurring in the chemocline water layer. Among our samples, *Chlorobia* were found only in the sediment of the meromictic lake (St. 6). During summer, anoxygenic phototrophic bacteria thrive in the chemocline zone of stratified lakes, where they carry out active photosynthesis. In winter, in ice-covered basins, where no light penetrates to the chemocline, *Chlorobia* precipitate to the surface of the sediment.

The third group comprised the families with significant relative abundance in the sediments of the Kanda and Kislaya Guba bays (stations 2–5), but they were absent or occurring in insignificant amounts at the reference St. 1 and St. 6. In the Kanda Guba sediments, there were numerous cyanobacteria of the genus *Cyanobium PCC-6307* (phylum *Cyanobacteria*, family *Cyanobiaceae*, order *Synechococcales*) (14.72 and 17.99% at St. 4 and St. 5, respectively). According to the abundance of cyanobacteria, marine St. 1 and Kislaya Guba stations 2 and 3 were oligotrophic (0.55, 0.08, and 0.31%, respectively), while sulfidic St. 6 (7.71%) was mesotrophic. Cyanobacteria perform oxygenic photosynthesis in the photic zone of freshwater and marine water bodies. Apart from the littoral zone, where light reaches the bottom, the sediment surface is the site of cyanobacterial accumulation, rather than of their habitation. Cyanobacterial abundance reflects the productivity of the photic zone; it depends mainly on the content of biogenic elements. Interestingly, *Cyanobium_PCC-6307* was the only cyanobacterial genus revealed in the studied sediments.

Uncultured archaea of the order *Woesearchaeales* (phylum *Nanoarchaeota*) were revealed in the sediments of stations 2–6 (5.7, 7.5, 7.0, 6.3, and 3.2%, respectively). In the sediment of marine St. 1, archaea of the phylum *Nanoarchaeota* were found in minor amounts (0.64%). Uncultured archaea of the order *Woesearchaeales* inhabit freshwater and weakly saline basins of the mesotrophic or eutrophic type; they are heterotrophs preferring microaerobic conditions [40].

Aerobic (microaerophilic) sulfur-oxidizing bacteria of the families *Desulfuromonadaceae* (phylum *Desulfobacterota*) and *Sulfurovaceae* (genera *Sulfuromonas* and *Sulfurovum*, phylum *Campilobacterota*) were found in the Kislaya Guba sediments (St. 3). These are typical gradient marine bacteria. They can oxidize elemental sulfur, sulfide, and other reduced sulfur species via aerobic or (for some species) nitrate respiration [41]. The known cultured members of the genus *Sulfurovum* are chemoorganotrophs that oxidize sulfur and thiosulfate, using oxygen or nitrate as electron acceptors [37]. Most members of this genus have been found in the samples of water and sediments from marine hydrothermal vents. However, *Sulfurovum* has also been found in Arctic seas [42].

Uncultured *Chloroflexi* of the family *Anaerolineaceae* (phylum *Chloroflexi*) were found in the sediments of stations 2–6 (up to 4.11% at St. 5). In the oxidized sediment of marine St. 1, they were considerably less abundant (0.65%). A significant part of the *Chloroflexi* was represented by uncultured heterotrophic bacteria of the genus *Caldilineaceae* SBR1031, class *Anaerolineae*. These bacteria are considered anaerobic syntrophs in mutualistic association with methanogenic archaea [43].

Sulfate-reducing bacteria of the phylum *Desulfobacterota* were found in all samples. Most revealed that *Desulfobacterota* belonged to the classes *Desulfobacteria* and *Desulfobulbia*. Uncultured sulfatereducers of the family *Desulfatiglandaceae* were found in reduced sediments of stations 4–6. In the sediments of marine St. 1, uncultured sulfate reducers of the genus *Desulfosarcina*, family *Desulfocapsaceae*, and *Desulfosarcinaceae*, g:SEEP-SRB1, forming associations with the ANME-1 archaea [44], were found. Sulfidogenesis by members of the phylum *Desulfobacterota* may occur as a result of S^0^ or sulfate reduction or of thiosulfate disproportionation [45]. These bacteria do not require organic substrates and grow as chemolithoautotrophs. In the studied Kanda Guba samples (Sts. 4, 5) and at St. 6, all these sulfate-reducing taxa were found, although no clearly dominant genera were revealed.

Archaea of the family SCGC_AAA011-D5 (*Nanoarchaeota* archaeon) were also found in the samples from Sts. 4–6.

Bacteria related to the family *Methylophilaceae* (phylum *Pseudomonadota*) were also found in the sediments of Sts. 4–6. Bacteria of this family have been isolated from the sediments of freshwater and brackish basins, soils, and rice fields. Cultured *Methylotenera* can utilize methylamine as the sole source of energy, carbon, and nitrogen [46]. These methanotrophs were associated with bacteria of the genus *Methyloceanibacter*, which reside in the sediments of northern seas and are capable of oxidizing a broad range of single-carbon compounds [47]. As was expected, detected methanotrophs of the family *Methylomonadaceae* were close to filamentous *gammaproteobacteria* related to *Crenothrix* sp. Some researchers consider them important methane consumers in stratified water basins [48].

## 4. Discussion

Tidal power stations are considered promising since it is traditionally thought that their operation has a minimal environmental effect. The world’s first TPS, Rance, was launched in 1966. Environmental safety was the main issue bothering the French society. Research established that the period of estuarine isolation during construction (5 years) was rather damaging to the aquatic environment [49]. However, partial stabilization of the flora and fauna, which changed somewhat under new environmental conditions, occurred after 10 years of operation [50].

The Annapolis experimental TPS (Canada) operated until 2019 and was shut down due to fish’s death caused by transfer through the turbines. The world’s largest TPS was built on Lake Sihwa (Republic of Korea) in the west of the Korean Peninsula. Lake Sihwa was formed in 1994 by constructing a dam across the Yellow Sea bay. As a result of the cessation of water exchange with the open part of the sea, stagnant waters with hydrogen sulfide contamination appeared [51]. The Jiangxia TPS (China) is situated in the Luoyuan Bay, where the tide reaches a maximum of 8.9 m.

At all TPSs listed above, investigations of their environmental effect have been carried out by hydrologists, fisheries experts, hydrobiologists, and ornithologists. Microbiological research has not been carried out.

Our study revealed that, according to their geochemical and biogeochemical parameters, the sediments of the Kislaya Guba and Kanda Bay, which have been artificially isolated from the major sea basins, retained the properties of marine sediments while becoming similar to the sediments of stratified basins (Table 1). These properties were, first of all, oxygen limitation or exhaustion, as well as a significant increase in the rates of reductive processes, resulting in sulfide and methane release. Microbial communities of the sediments of isolated marine reservoirs change their composition significantly compared to the original composition of sediments of flowing sea bays. Changes in the rates of the key microbial processes were objective indicators of these events. In the sediments of the Kanda and Kislaya Guba bays, the rates of CO_2_ assimilation, sulfate reduction, methane oxidation, and hydrogenotrophic methanogenesis increased (Table 1). Thus, the hydrochemical and biogeochemical state of the Kanda and Kislaya Guba bays changed, indicating a shift to a stratified basin with sulfide-containing, oxygen-free near-bottom layers. Such basins are known at the White Sea coast [18]. Anoxic zones of stratified water bodies have long been of interest to microbiologists as relict ecosystems [10,21,52,53,54,55]. Stable anoxic zones exist in the meromictic Lake Mogil’noe (Kildin Island, Russia), Framvaren Fjord (Southern Norway), and the Black Sea [56,57,58]. In two deep-water depressions of the Caspian Sea, the anoxic regime is less stable [59]. Numerous small, stratified basins (marine-derived lakes) are known at the coast of the Kandalaksha Gulf, White Sea. These water bodies are of unique origin since they were formed by separation from the sea due to rapid land elevation (~40 cm in the recent 100 years) [60]. The washout regime weakens with increasing distance from the sea, resulting in extensive sedimentogenesis, higher rates of anaerobic microbial processes (especially sulfate reduction), and sulfide release into the monimolimnion [61]. Sulfate-reducing bacteria are not the only anaerobic microorganisms involved in the transformation of organic compounds in such environments. Together with sulfate-reducing bacteria, methanogenic archaea, responsible for methane formation under anoxic conditions, are involved in the terminal phase of organic matter decomposition.

Our studies showed that in the rich community of the upper sediments, it was possible to determine the microorganisms acting as indicators of the changes resulting in anoxic zone formation. These are archaea of the genera *Methanoregula* and *Methanosaeta*, while archaea of the genus *Nitrosopumilus* (phylum *Crenarchaeota*) may be termed anti-indicator microorganisms since they occur only in strongly oxidized sediments of the open sea. Microorganisms involved in the sulfur cycle were also good indicators of stagnant seawater. While they were not numerous in the water column of the Kanda and Kislaya Guba bays, they are potentially good indicators since they were not revealed at marine St. 1. These organisms are anoxygenic phototrophs of the class *Chlorobia* (*Chlorobium phaeovibrioides*, *Pelodictyon phaeoclathratiforme*).

**Figure 5:**
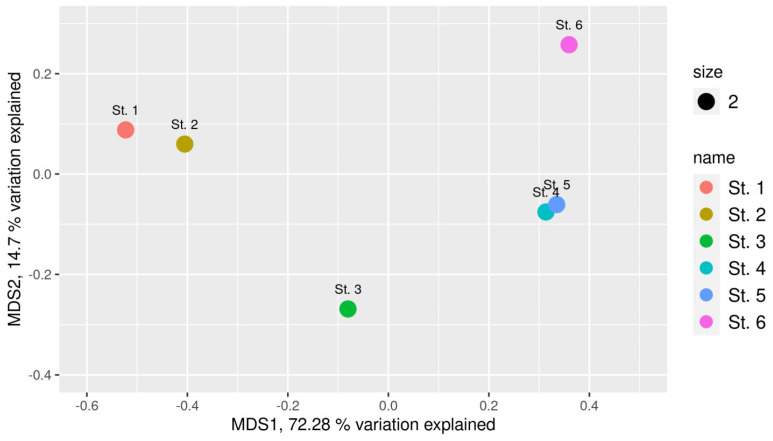
nMDS plot of the Bray–Curtis dissimilarity index of samples. Each circle represents a different sample. Site designations: St.1, marine sediment; St. 2, Kislaya Guba tidal power station bay, sediment 0–2 cm; St. 3, Kislaya Guba tidal power station bay, sediment 2–7 cm; St. 4, Kanda Bay, apex part, sediment 0–2 cm; St. 5, Kanda Bay, sediment 0–2 cm; St. 6, meromictic lake Trekhtzvetnoe, sediment 0–2 cm.

Importantly, our studies showed reversibility of the processes converting a flow sea bay into a stratified basin with an anoxic near-bottom water layer. The microbial community of the surface sediment layer at St. 2 (oxidized, Figure 2), accumulated after partial opening of the sluice gates, turned out to be similar to the community of the marine sediment (St. 1) (Figure 5).

The microbial community of the subsurface sediment (St. 3) comprised numerous microorganisms inhabiting reduced sulfidic sediments (St. 6) while retaining the microorganisms of the open-sea sediments. Microbial communities of the weakly reduced Kanda Bay sediments (Sts. 4 and 5) were similar to those of St. 6, although their species diversity was significantly higher.

Our research showed that in the upper sediments of marine bays partially isolated from the main basin, the microbial taxonomic composition is determined by the flow (water exchange) in these reservoirs. The exploitation of tidal power stations implies the possibility of regulating the water flow through the dam in both directions. The optimal regime of the free flow of the tidal water will prevent the development of the stagnant, sulfide-containing near-bottom water layer in isolated basins. The upper sediment microbial community may act as an indicator of unfavorable reductive processes.

## Figures and Tables

**Table 1:** Physicochemical and biogeochemical characteristics of the samples of bottom sediments and near-bottom water from the Kislaya Guba (Sts. 2 and 3) and Kanda bays (Sts. 4 and 5), marine part of the sea gulf (St. 1), and the meromictic, sulfide-containing Lake Trekhtzvetnoe (St. 6).

Station	St. 1. “Marine,” Marine Sediment, White Sea Gulf	St. 2. Kislaya Guba, Tidal Power Station Bay	St. 3. Kislaya Guba, Tidal Power Station Bay	St. 4. Kanda Guba, Apex Reach	St. 5. Kanda Guba, Marine Reach	St. 6. “Anoxic,” Meromictic Lake Trekhtzvetnoe
Coordinates	67°07′25″ N 32°15′42″ E	69°21′90″ N 33°04′12″ E	69°21′58″ N 33°03′98″ E	67°08′23″ N 31°53′20″ E	67°08′88″ N 32°10′70″ E	66°43′01″ N, 32°51′28″ E
Depth, m	28	34	35	18	21.5	7.0
Sediment layer	0–2 cm	0–2 cm	2–7 cm	0–2 cm	0–2 cm	0–2 cm
Sediment appearance	Liquid, with gray sand and aleurite	Brown, loose, flaky	Black, pelitic, homogeneous	Liquid, black, sulfide-containing	Liquid, black, sulfide-containing	Liquid, black, sulfide-containing
T_bottom_, °C	1.3	1.4	1.4	4.0	4.1	5.1
Salinity, ‰	24	33.2	33.2	13.5	22.0	21.9
Eh, mV	220	20	+40 − 30	−390	−340	−420
O_2_, * mmol L^−1^	0.36	0.15	0	0	0	0
H_2_S mmol dm^−3^	0	0	0	0.46	0.85	up to 13.25
CH_4_ mmol dm^−3^	0.64	12.4–15.8	12–28	45–80	124–158	up to 2220
MGh, µmol dm^−3^ day^−1^	˂2.0 × 10^−3^	3–6 × 10^−3^	8–10 × 10^−3^	12–40 × 10^−3^	8–24 × 10^−3^	90–130 × 10^−3^
DCA, µmol dm^−3^ day^−1^	0.9–1.1	1.4–4.0	1.2–3.8	3–5	5–7	48–122
SR µmol S dm^−3^ day^−1^	0.05–0.15	0.5–1.2	0.3– 0.9	8–14	12–20	68
MO, µmol dm^−3^ day^−1^	0.05–0.09	0.25–0.31	0.1–0.17	0.17–0.41	0.08–0.19	1.8
MA, 10^6^ cells mL^−1^/B, µg L^−1^	0.20/78	0.48/182	0.48/182	0.52 195	0.42/170	12/4800

* Oxygen concentration and total microbial abundance (MA) were determined in near-bottom water collected immediately above the sediment.

**Table 2:** Alpha diversity indices in the sediment samples from stations 1–6 in the Kanda and Kislaya Guba bays, Kandalaksha Gulf (White Sea), and the meromictic Lake Trekhtzvetnoe.

Sediment Sample	Archaea OTU %	Bacteria OTU %	Chao1	Shannon *H’* (Log with Base *e*)
St. 1. Marine sediment	27.98	71.40	789.5	4.63
St. 2. Kislaya Guba, tidal power station bay, 0–2 cm	39.80	54.6	1425.9	5.08
St. 3. Kislaya Guba, tidal power station bay, 2–7 cm	13.80	74.02	1606.2	5.98
St. 4. Kanda Guba, apex part	20.30	65.40	1471.1	5.68
St. 5. Kanda Guba, Fedoseevskiy reach	18.83	68.83	1568.3	5.68
St. 6. Meromictic Lake Trekhcvetnoe	23.85	71.04	873.5	5.29

## Data Availability

The original contributions presented in this study are included in the article. Further inquiries can be directed to the corresponding authors.
